# Association of Blood Mercury Levels with the Risks of Overweight and High Waist-to-Height Ratio in Children and Adolescents: Data from the Korean National Health and Nutrition Examination Survey

**DOI:** 10.3390/children8121087

**Published:** 2021-11-25

**Authors:** Ky Young Cho

**Affiliations:** Department of Pediatrics, Hallym University Kangnam Sacred Heart Hospital, Seoul 07441, Korea; choky96@hallym.or.kr; Tel.: +82-2-829-5142

**Keywords:** mercury, overweight, risk, adolescent, Korean national health and nutrition examination survey

## Abstract

A previous study in adults demonstrated the substantial role of mercury exposure in the development of overweight and obesity. Although children and adolescents are more susceptible to the toxic effects of mercury than adults, studies on the association of overweight and obesity with mercury exposure is limited. This study aimed to investigate the association of blood mercury levels with the body mass index (BMI) and waist-to-height ratio (WHtR) as obesity indices in Korean children and adolescents. The analyzed cross-sectional data were obtained from 1327 participants (age: 10–18 years; 672 male and 655 female) who completed the Korean National Health and Nutrition Examination Survey 2010–2013. The covariates included sociodemographic factors (age, sex, and household income), dietary factors (fish, shellfish, and seaweed consumption), lifestyle factors (alcohol consumption, smoking status, and exercise), and blood hematocrit levels. The adjusted geometric mean blood mercury level was 2.19 µg/L, and the level of mercury was significantly higher in the overweight (BMI ≥ 85th gender and age-specific percentiles) and high WHtR (cutoff: ≥0.5) groups than in the normal group. In all the participants, the blood mercury levels were significantly positively associated with the BMI and WHtR after adjusting for all covariates (*p* < 0.05). All the participants in the highest blood mercury level quartile were at a higher risk for overweight and a high WHtR than those in the lowest quartile after adjusting for all covariates (*p* < 0.05). Our study suggests a significant association between mercury exposure and the risks of overweight and high WHtR in Korean children and adolescents.

## 1. Introduction

The prevalence of overweight and obesity in children and adolescents has increased over the past decade [1]. Overweight and obesity in children and adolescents lead to critical health conditions, including hypertension, type 2 diabetes mellitus, cardiovascular complications, and psychological problems, which can continue into adulthood [2]. An imbalance between caloric intake and physical activity has been considered a principal cause of overweight and obesity [3]. Various factors affect overweight and obesity, including genetic, lifestyle, cultural, and environmental factors [4]. Many studies have demonstrated a substantial role of environmental factors in the development of overweight and obesity [5]. Several studies have reported that bisphenol A exposure is associated with childhood obesity, but those results have been controversial [6,7]. Demonstrating the actual contribution of environmental factors to obesity is challenging because of the difficulties in proving a causal association. These difficulties could lead to an underestimation of environmental factors as a risk factor for obesity.

Among environmental factors, mercury exposure is associated with obesity. A study of 200 healthy adults aged 30 to 64 years showed a significant association between blood mercury levels and visceral adipose tissue [8]. In a study of 2114 adults, significant relationships were also demonstrated between blood mercury levels and the body mass index (BMI) and waist circumference (WC) [9]. However, another study reported no significant relationships between blood mercury levels and the adipose tissue content [10]. Additionally, studies on U.S. non-pregnant adults and children have revealed that blood mercury levels are inversely related to the BMI [11]. Thus, previous findings on the association of mercury exposure with overweight and obesity are inconsistent.

Mercury generally exists in the environment, and most elemental and inorganic mercury exposure occurs by breathing air containing elemental mercury vapor in some occupations or through devices containing mercury, such as thermostats or thermometers [12,13]. In the human body, 80 to 90% of organic mercury intake is attributed to fish and shellfish intake, and 75 to 90% of organic mercury existing in fish and shellfish is in the form of methylmercury, which shows the strongest toxicity in humans [14]. Long-term exposure to high levels of methylmercury primarily causes nervous system effects, including disturbances in vision, hearing, and speech, tingling and numbness in the fingers and toes, a lack of coordination, and muscle weakness [12,15]. Not only a high level of exposure but also relatively low-level exposure to mercury is related to an increased risk of cognitive impairment, hypertension, alteration of heart function, and renal dysfunction in children and adults [16]. Children and adolescents are more susceptible to the toxic effect of mercury than adults because their nervous systems are still developing and may be more vulnerable [17]. Various risk factors, such as age, sex, genetics, and culture, are associated with elevated blood mercury levels [18]. Obesity can also be associated with elevated blood mercury levels; however, studies on the relationship between obesity and the blood mercury status in children and adolescents are insufficient.

The BMI is a conventionally used obesity index for population-based screening. However, the BMI is a rather poor indicator of body fat because it reflects not only adiposity but also muscle mass [19]. Many studies have investigated alternatives to the BMI that reflects body fat related to metabolic risk factors. Current studies have shown that the waist-to-height ratio (WHtR) is superior to the BMI, WC, and waist-to-hip for predicting metabolic risk factors [20]. This study aimed to investigate the association of blood mercury levels with the body mass index (BMI) and waist-to-height ratio (WHtR) as obesity indices in Korean children and adolescents using data from the Korean National Health and Nutrition Survey (KNHANES) 2010–2013.

## 2. Materials and Methods

### 2.1. Study Design

**Figure 1:**
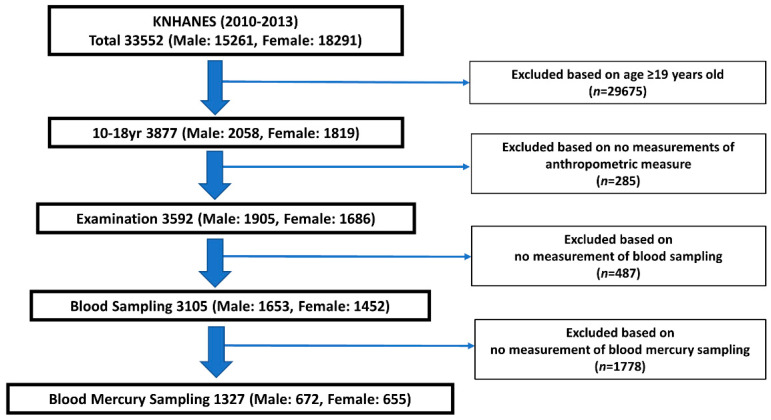
Study Participants.

The KNHANES is a series of nationally representative population-based cross-sectional surveys concerning health and nutritional status that have been conducted using a stratified, multistage, and probability sampling design by the Korea Disease Control and Preventive Agency (KDCA) [21]. Our study used data from the questionnaire, physical examination components, laboratory indices, and diet assessment of the KNHANES V 1-3 and VI-1 (2010–2013). Among an initial enrolled sample of 15,261 men and 18,291 women, we excluded those who were aged younger than 10 years and older than 19 years, had no data on anthropometric measures, and had no measurement data on blood mercury levels (Figure 1). The final number of participants was 1327 (10–18 years of age; 672 male and 655 female), and the participants were divided into normal and overweight groups. Overweight was defined as a BMI ≥ 85th gender and age-specific percentiles based on the 2017 Korean growth chart [22]. The remaining participants comprised the normal group. The BMI was defined as the participants’ weight in kilograms divided by the height in meters squared (kg/m^2^). The WHtR was defined as the participant’s WC in cm divided by the height in cm. The participants were additionally divided into male and female groups.

### 2.2. Ethics Approval and Consent to Participate

This study was conducted according to the guidelines of the Declaration of Helsinki. The KNHANES was approved by the Institutional Review Board of the KDCA (2010-02CON-21-C, 2011-02CON-06-C, 2012-01EXP-01-2C, and 2013-07CON-03-4C) and comprises a health interview, health examination survey, and nutrition survey. All the surveys were conducted with the participants’ consent, and data from the KNHANES were obtained in a fully anonymized and deidentified manner. Therefore, this study was exempt from the requirement for approval by the Hallym University Kangnam Sacred Heart Hospital Institutional Review Board under IRB No. 2021-11-006 (10 November 2021). Patient consent for this study was waived because of the retrospective nature of the study and analysis of anonymous clinical data.

### 2.3. Measurements of the Blood Mercury Levels in Whole Blood

To measure the levels of mercury in whole blood, 3-mL blood samples were obtained from the participants after an 8-h overnight fast. The blood mercury levels were measured using the gold amalgam collection method and the Direct Mercury Analyzer 80 (DMA-80; Milestone, Bergamo, Italy) at the Neodin Medical Institute (Seoul, South Korea), a central laboratory certified by the Korean Ministry for Health and Welfare [23]. For internal quality assurance and control, commercial reference material was used (Lyphochek^®^ Whole Blood Metals Control; Bio-Rad, Hercules, CA, USA) with coefficients of variation of 1.59–4.86% among four reference samples. For external quality assurance and control, the Neodin Medical Institute was approved by the German External Quality Assessment at Friedrich-Alexander University and by the Quality Assurance Program at the Korea Occupational Safety and Health Agency. The limit of detection for blood mercury using this method was 0.158 μg/L, and no participants presented a blood mercury concentration below this level in this study. Blood mercury levels were categorized into quartiles (Qs) and stratified by sex and age. Q4 of blood mercury levels was defined as the highest value.

### 2.4. Determination of Covariates

Data were obtained from the questionnaires concerning sociodemographic, lifestyle, and dietary factors from the KNHANES. Household income was determined according to the quartile values of equalized household income (total household income divided by the square root of the number of household members) [24]. The current alcohol consumption status was classified as drinker or nondrinker, defined according to alcohol consumption more than once in the last month. The current smoking status was classified as smokers or nonsmokers, defined according to smoking more than once in the last month. The current exercise status was classified based on whether the participants had engaged in strenuous exercise in the previous week. The total amount of seafood (fish, shellfish, and seaweed) consumed within the previous 24 h was obtained using a 24-h dietary recall questionnaire administered by a trained nutritionist. Additionally, the hematocrit was used as a covariate and categorized into quartiles. For all quartile categorizations, Q4 was defined as the highest value for each variable.

### 2.5. Statistical Analysis

Statistical analyses were performed using SPSS statistical software (version 22; IBM Co., Chicago, IL, USA) and visualized using the R program (version 3.6.2). The complex weighted sample descriptive procedure was used to evaluate the numerical variables, and the complex weighted sample crosstabs procedure was used to evaluate the categorical or ordinal variables using weights provided by the KNHANES. The complex weighted samples general linear model was used to calculate the geometric means and 95% confidence intervals (CIs) to compare the blood mercury levels between the groups of study variables. The regression coefficient (95% CIs) of the association among the BMI, WHtR, and blood mercury levels was calculated using the complex weighted samples general linear model after controlling the covariates, including sociodemographic factors (age, sex, household income), dietary factors (seafood consumption), lifestyle factors (alcohol consumption, smoking status, and exercise), and blood hematocrit levels. Complex weighted-samples logistic regression analysis was used to determine the odds ratios (ORs) and 95% CIs to predict the risk of overweight and a high WHtR, adjusted for the same covariates. Participants with blood mercury levels in Q1 were considered the reference group. Complex weighted-samples logistic regression analysis was used to obtain the *p* value for the trend in the overweight and high WHtR groups across increasing blood mercury quartiles. A receiver operating characteristic (ROC) curve was constructed to evaluate the performance of the blood mercury levels to predict overweight and a high WHtR. A two-tailed *p* value < 0.05 was considered statistically significant.

## 3. Results

### 3.1. Characteristics of the Participant Subsection

This cross-sectional survey was conducted among 1327 participants (672 male and 655 female) who had completed the KNHANES, 2010–2013. The characteristics of the participants are presented in Table 1. The number of participants in the overweight group was 267 (20.1%); in the normal group, the number was 1060 (79.9%). As expected, weight, the BMI, WC, and the WHtR were significantly higher in the overweight group than in the normal group among all, male, and female participants (*p* < 0.05; Table 1). The overweight group showed significantly higher geometric mean blood mercury levels than the normal group among all, male, and female participants (*p* < 0.01; Table 1). The overweight and normal groups did not differ in the intake of seafood in the previous 24 h, except in the subgroup of female participants (*p* < 0.05; Table 1). No significant differences were identified in age, the blood hematocrit levels, proportions of participants in 3-year interval age groups, survey year, household income quartiles, alcohol consumption, or smoking status between the normal and overweight groups (Table 1). No significant differences were found in exercise between the normal and overweight groups of all participants and the female subgroup, but not the male subgroup (Table 1). Among all the participants, male participants reported more frequent engagement in exercise than female participants (male: 391 [71.7%]; female 234 [45.2%]; *p* < 0.001). Among all the participants, the WC in the male group was significantly higher than that in the female group (male: 71.62 ± 0.48; female: 67.75 ± 0.41; *p* < 0.001). The blood hematocrit levels in the male group were significantly higher than those in the female group (male: 43.44 ± 0.15; female: 39.89 ± 0.12; *p* < 0.001).

### 3.2. Blood Mercury Levels according to the Participants’ General Characteristics

Table 2 presents the geometric means and 95% CIs of blood mercury levels according to the participants’ general characteristics. The geometric means (95% CIs) of blood mercury levels in all, male, and female participants were 2.19 (2.06–2.26), 2.18 (1.98–2.38), and 2.14 (1.91–2.36) µg/L, respectively, after controlling for age, sex, total intake of seafood in the previous 24 h, household income, alcohol consumption, smoking status, and the blood hematocrit levels. No significant differences were found in the crude or adjusted blood mercury levels between the male and female groups. A WHtR cutoff point of 0.5 is generally accepted as a screening tool for metabolic syndrome risks independent of gender and ethnicity in children, adolescents, and adults [25]. The WHtR group was divided into high (≥0.5) and normal (<0.5) groups. The geo-mean of blood mercury levels was significantly higher in the overweight and high WHtR groups than in the normal group among all the participants after adjusting for covariates (*p* < 0.05; Table 2). The Q3 and Q4 blood hematocrit levels showed significantly higher crude and adjusted geo-mean blood mercury levels than the Q1 blood hematocrit levels in all the participants and the male group (*p* < 0.05; Table 2). Q4 of the total amount of seafood consumed in the previous 24 h showed significantly higher crude and adjusted blood mercury levels than Q1 among all, male, and female participants (*p* < 0.05; Table 2). No significant differences were found in the blood mercury levels according to the current alcohol consumption, smoking status, and exercise among all the participants and the male subgroup (Table 2).

### 3.3. Associations among the BMI, WHtR, and Blood Mercury Levels

**Figure 2:**
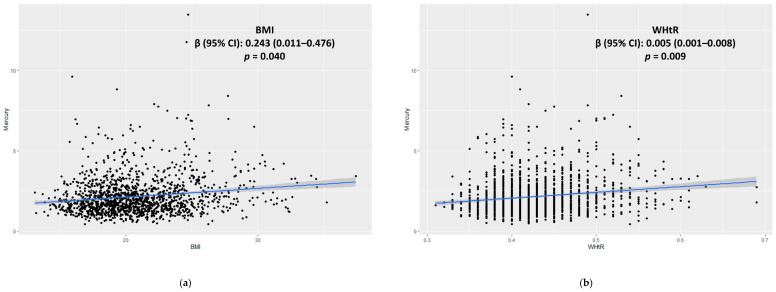
Scatter Plot of the Positive Association among the BMI (**a**), WHtR (**b**), and Blood Mercury Concentrations in All the Participants. Abbreviations: BMI, body mass index; WHtR, waist to height ratio.

The blood mercury levels were significantly positively associated with the BMI in the unadjusted and adjusted models of all the participants (regression coefficients, β [95% CIs]: 0.24 [0.01–0.48]; *p* = 0.04; Table 3; Figure 2a). In the male and female subgroups, the model adjusted for age and sex showed significantly positive associations between the blood mercury levels and BMI (β [95% CIs]: 0.43 [0.14–0.71] and 0.47 [0.16–0.79]; *p* = 0.004 and 0.003, respectively; Table 3). Blood mercury levels were significantly positively associated with the WHtR in the unadjusted and adjusted models of all the participants (β [95% CIs]: 0.005 [0.001–0.008]; *p* = 0.009; Table 3; Figure 2b). In the female subgroup, the model adjusted for all the variables showed significantly positive associations between the blood mercury levels and WHtR (β [95% CIs]: 0.005 [0.001–0.007]; *p* = 0.016; Table 3; Figure 2b).

### 3.4. Logistic Regression and ROC Curve Analysis for the Overweight and High WHtR Groups

**Figure 3:**
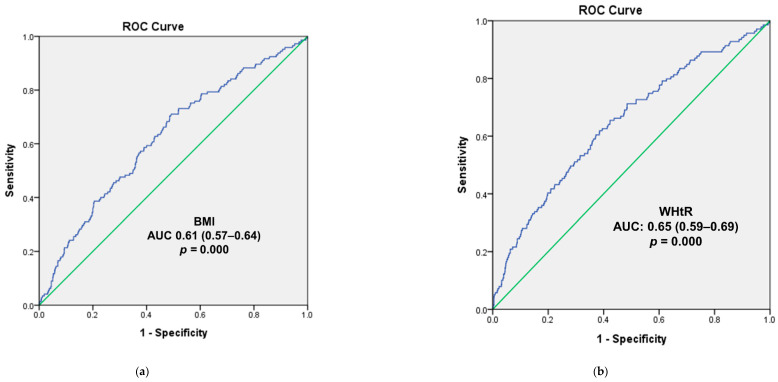
Receiver Operating Characteristic (ROC) Curve of the Blood Mercury Concentrations to Predict Overweight (**a**) and the High Waist-to-Height Ratio (**b**) in All the Participants. Abbreviations: BMI, body mass index; WHtR, waist to height ratio; AUC, area under the receiver operating characteristic curve.

All the participants and the male subgroup in the highest blood mercury level quartile were at a higher risk of overweight than those in the lowest quartile after adjusting for all covariates (ORs [95% CIs]: 1.93 [1.07–3.46] and 2.36 [1.06–5.29]; *p* value = 0.03 and 0.04, respectively; Table 4). In the female subgroup, participants in the highest blood mercury level quartile were at a higher risk of overweight than those in the lowest quartile after adjustment for age and sex (ORs [95% CIs]: 2.41 [1.23–4.71]; *p* value = 0.01, Table 4). In all the participants and the male and female subgroups, the complex weighted-samples logistic regression analysis showed that the risk of overweight increased as the blood mercury level quartile increased after adjusting for age and sex (ORs [95% CIs]: 1.32 [1.13–1.55], 1.26 [1.02–1.56], and 1.42 [1.12–1.80], *p* for trend = 0.001, 0.03, and 0.004, respectively; Table 4). All the participants and the male subgroup in the highest blood mercury level quartile were at a higher risk of a high WHtR than those in the lowest quartile, after adjusting for all covariates (ORs [95% CIs]: 3.00 [1.15–7.84] and 4.77 [1.35–16.89]; *p* value = 0.025 and 0.016, respectively; Table 4). The ROC analysis of blood mercury levels to predict overweight in all the participants showed that the area under the curve (AUC) was 0.61 (95% CIs: 0.57–0.64; *p* value = 0.000; Figure 3a). The ROC analysis of blood mercury levels to predict a high WHtR in all the participants showed that the AUC was 0.65 (95% CIs: 0.59–0.69; *p* = 0.000; Figure 3b).

**Table 1:** Characteristics of the Participants.

	All (*n* = 1327)			Male (*n* = 672)			Female (*n* = 655)		
	Normal (*n* = 1060)	Overweight (*n* = 267)	*p* Value	Normal (*n* = 529)	Overweight (*n* = 143)	*p* Value	Normal (*n* = 531)	Overweight (*n* = 124)	*p* Value
Age (yrs.)	14.33 ± 0.09	14.19 ± 0.18	0.48	14.37 ± 0.13	14.02 ± 0.25	0.20	14.29 ± 0.14	14.40 ± 0.27	0.71
Age group			0.55			0.30			
10–12 yrs.	360 (27.8)	93 (28.3)		182 (27.6)	53 (31.3)		178 (28.1)	40 (24.6)	0.80
13–15 yrs.	380 (34.3)	96 (37.8)		194 (34.3)	50 (38.7)		186 (34.3)	46 (36.6)	
16–18 yrs.	320 (37.9)	78 (33.9)		153 (38.1)	40 (29.9)		167 (37.6)	38 (38.8)	
Year			0.49			0.55			0.55
2010	266 (25.8)	71 (24.1)		125 (24.9)	43 (26.7)		141 (26.8)	28 (21.0)	
2011	266 (26.1)	72 (28.7)		140 (26.9)	33 (26.4)		126 (25.2)	39 (31.5)	
2012	277 (24.8)	50 (20.4)		141 (25.7)	27 (19.4)		136 (23.8)	23 (21.5)	
2013	251 (23.3)	74 (26.8)		123 (22.6)	40 (27.4)		128 (24.2)	34 (26.0)	
Weight (kg)	51.81 ± 0.37	70.77 ± 0.98	0.00 *	54.53 ± 0.58	75.17 ± 1.54	0.00 *	48.80 ± 0.40	65.39 ± 0.98	0.00 *
Height (cm)	161.88 ± 0.39	163.54 ± 0.71	0.05	165.58 ± 0.63	166.78 ± 1.09	0.34	157.78 ± 0.38	159.59 ± 0.75	0.03 *
BMI (kg/m^2^)	19.58 ± 0.08	26.19 ± 0.21	0.00 *	19.66 ± 0.12	26.69 ± 0.26	0.00 *	19.48 ± 0.10	25.60 ± 0.34	0.00 *
WC (cm)	66.50 ± 0.08	82.19 ± 0.59	0.00 *	67.78 ± 0.35	85.38 ± 0.84	0.00 *	65.08 ± 0.31	78.28 ± 0.73	0.00 *
WHtR	0.41 ± 0.00	0.50 ± 0.00	0.00 *	0.41 ± 0.00	0.51 ± 0.00	0.00 *	0.41 ± 0.00	0.49 ± 0.00	0.00 *
Mercury (µg/L)	2.09 ± 0.04	2.43 ± 0.08	0.00 *	2.13 ± 0.05	2.46 ± 0.10	0.01 *	2.05 ± 0.06	2.39 ± 0.11	0.01 *
Hematocrit (%)	41.66 ± 0.12	41.93 ± 0.23	0.30	43.28 ± 0.16	43.49 ± 0.33	0.55	39.87 ± 0.14	40.01 ± 0.21	0.56
Seafood consumption in the previous 24 h (g/day)	54.18 ± 3.27	59.31 ± 5.69	0.44	63.67 ± 5.49	52.76 ± 6.86	0.22	43.68 ± 3.16	67.68 ± 9.35	0.02 *
Household income			0.42			0.15			0.22
Quartile 1	126 (14.6)	29 (12.7)		61 (15.0)	12 (11.0)		65 (14.1)	17 (14.8)	
Quartile 2	274 (29.9)	82 (34.1)		137 (30.6)	37 (29.3)		137 (29.2)	45 (39.7)	
Quartile 3	342 (29.5)	71 (24.5)		172 (28.4)	35 (22.3)		170 (30.7)	36 (27.1)	
Quartile 4	311 (26.0)	83 (28.7)		156 (26.1)	57 (37.4)		155 (26.0)	26 (18.3)	
Consuming alcohol more than once in the last month			0.32			0.57			0.40
No	1012 (95.3)	253 (93.1)		504 (94.0)	133 (92.3)		508 (96.8)	120 (79.3)	
Yes	39 (4.7)	11 (6.9)		22 (6.0)	9 (7.7)		17 (3.2)	2 (6.0)	
Smoking more than once in the last month			0.38			0.68			0.38
No	979 (93.7)	239 (91.8)		478 (91.1)	124 (89.7)		501 (96.7)	115 (94.4)	
Yes	50 (6.3)	18 (8.2)		35 (8.9)	12 (10.3)		15 (3.3)	6 (5.6)	
Exercise in the last week			0.07			0.02 *			0.90
Yes	489 (57.7)	136 (65.9)		303 (68.8)	88 (82.5)		186 (45.0)	48 (45.8)	
No	334 (42.3)	63 (34.1)		114 (31.2)	18 (17.5)		220 (55.0)	45 (54.2)	

The values are presented as mean ± standard error or number (%). *p* value by *t*-test or chi-squared test. Abbreviations: BMI, body mass index; WC, waist circumference; WHtR, waist to height ratio. * *p* < 0.05.

**Table 2:** Geometric Means (95% CIs) of the Blood Mercury Concentrations (µg/L) According to the Participants’ General Characteristics.

	All			Male			Female		
	*n*	Crude	Adjusted (*n* = 1096)	*n*	Crude	Adjusted (*n* = 553)	*n*	Crude	Adjusted (*n* = 543)
All	1327	2.16 (2.09–2.24)	2.19 (2.06–2.26)	672	2.13 (2.02–2.24)	2.18 (1.98–2.38)	655	2.05 (1.92–2.17)	2.14 (1.91–2.36)
Age group	1327			672			655		
10–12 yrs.		2.09 (1.99–2.19)	2.31 (1.95–2.67)		2.09 (1.95–2.24)	2.39 (1.96–2.82)		2.08 (1.96–2.20)	2.21 (1.58–2.84)
13–15 yrs.		2.23 (2.11–2.36)	2.27 (2.03–2.52)		2.33 (2.16–2.50)	2.30 (2.08–2.53)		2.12 (1.96–2.28)	2.17 (1.69–2.66)
16–18 yrs.		2.15 (2.02–2.28)	1.96 (1.59–2.33)		2.16 (2.02–2.31)	1.73 (1.31–2.16)		2.14 (1.92–2.36)	2.25 (1.66–2.84)
Overweight Group	1327			672			655		
Normal		2.09 (2.01–2.17)	2.07 (1.89–2.26)		2.13 (2.02–2.24)	2.06 (1.87–2.25)		2.05 (1.92–2.17)	2.15 (1.77–2.53)
Overweight		2.43 (2.28–2.58) **	2.34 (2.08–2.59) **		2.46 (2.25–2.66) **	2.31 (2.04–2.58) *		2.39 (2.17–2.62) **	2.35 (1.90–2.81)
WHtR Group	1296			660			636		
Normal		2.11 (2.04–3.29)	2.14 (1.93–2.35)		2.14 (2.04–2.24)	2.16 (1.97–2.35)		2.08 (1.97–2.20)	2.22 (1.78–2.66)
High		2.53 (2.28–2.77) **	2.49 (2.11–2.87)*		2.51 (2.23–2.78) *	2.38 (2.04–2.73)		2.57 (2.07–3.07) *	2.73 (1.87–3.60)
Hematocrit	1327			672			655		
Quartile 1		1.99 (1.86–2.14)	1.98 (1.74–2.22)		1.85 (1.67–2.03)	1.73 (1.45–2.01)		2.04 (1.87–2.21)	2.13 (1.71–2.55)
Quartile 2		2.15 (2.02–2.28)	2.14 (1.88–2.39)		2.14 (1.93–2.34) *	1.97 (1.69–2.24)		2.16 (1.99–2.33)	2.31 (1.85–2.76)
Quartile 3		2.22 (2.09–2.34) *	2.25 (2.01–2.49) *		2.25 (2.09–2.41) **	2.24 (1.99–2.49) **		2.17 (1.96–2.38)	2.28 (1.84–2.73)
Quartile 4		2.27 (2.13–2.41) **	2.24 (1.99–2.45) *		2.28 (2.13–2.43) **	2.21 (1.98–2.45) **		2.16 (1.77–2.54)	2.37 (1.77–2.97)
Household Income	1318			667			651		
Quartile 1		2.12 (1.97–2.27)	2.11 (1.86–2.35)		2.11 (1.90–2.31)	2.08 (1.83–2.34)		2.13 (1.92–2.36)	2.20 (1.73–2.67)
Quartile 2		2.03 (1.91–2.15)	2.01 (1.78–2.25)		1.98 (1.84–2.11)	1.92 (1.70–2.13)		2.09 (1.89–2.28)	2.17 (1.72–2.62)
Quartile 3		2.16 (2.04–2.28)	2.12 (1.88–2.35)		2.24 (2.07–2.41)	2.14 (1.88–2.39)		2.07 (1.91–2.23)	2.16 (1.73–2.59)
Quartile 4		2.35 (2.19–2.52) *	2.34 (2.06–2.61)		2.46 (2.23–2.68) *	2.34 (2.05–2.63)		2.21 (1.96–2.47)	2.34 (1.86–2.83)
Seafood Consumption	1137			576			561		
Quartile 1		1.99 (1.87–2.13)	1.98 (1.77–2.20)		2.09 (1.91–2.27)	2.02 (1.78–2.26)		1.91 (1.71–2.10)	1.96 (1.55–2.38)
Quartile 2		2.13 (1.97–2.29)	2.10 (1.85–2.36)		2.15 (1.94–2.37)	2.04 (1.77–2.31)		2.10 (1.85–2.26)	2.18 (1.72–2.64)
Quartile 3		2.19 (2.06–2.32) *	2.14 (1.90–2.37)		2.26 (2.04–2.47)	2.10 (1.84–2.37)		2.13 (1.97–2.29)	2.21 (1.77–2.64) *
Quartile 4		2.38 (2.22–2.54) **	2.33 (2.04–2.61) *		2.34 (2.17–2.51) *	2.29 (2.05–2.53) *		2.44 (2.13–2.75) **	2.49 (2.02–2.96) **
Alcohol consumption more than once in the last month	1315			668			647		
No		2.16 (2.09–2.24)	2.19 (2.04–2.33)		2.21 (2.11–2.31)	2.21 (2.06–2.36)		2.12 (2.01–2.23)	2.22 (1.87–2.56)
Yes		2.17 (1.92–2.42)	2.09 (1.75–2.45)		2.15 (1.92–2.38)	2.03 (1.72–2.34)		2.20 (1.63–2.77)	2.23 (1.56–2.89)
Smoking during the last month	1021			649			637		
No		2.17 (2.09–2.24)	2.16 (1.99–2.32)		2.21 (2.10–2.31)	2.15 (1.99–2.30)		2.13 (2.02–2.24)	2.16 (1.85–2.48)
Yes		2.13 (1.91–2.35)	2.13 (1.81–2.45)		2.15 (1.95–2.36)	2.09 (1.80–2.39)		2.06 (.146–2.66)	2.28 (1.59–2.97)
Exercise in the last week	1022			523			499		
Yes		2.22 (2.12–2.33)	2.19 (1.98–2.41)		2.23 (2.11–2.35)	2.15 (1.96–2.34)		2.21 (2.01–2.40)	2.36 (1.90–2.84) *
No		2.09 (1.97–2.22)	2.11 (1.87–2.35)		2.19 (1.99–2.41)	2.22 (1.95–2.48)		2.03 (1.87–2.18)	2.09 (1.65–2.55) *

The values are presented as geomeans (95% confidence intervals). * *p* < 0.05, ** *p* < 0.01 by *t*-test. Adjusted for age, sex, seafood consumption, household income, smoking, drinking, and blood hematocrit levels. Abbreviations: Seafood: fish, shellfish, and seaweed.

**Table 3:** Regression Coefficients (β) and 95% Confidence Intervals for the Body Mass Index and Waist to Height Ratio Associated with Blood Mercury Concentrations, Stratified by Age and Sex.

**Body Mass Index**									
	***n***	**All (*n* = 1327)**	***p* Value**	***n***	**Male (*n* = 672)**	***p* Value**	***n***	**Female (*n* = 655)**	***p* Value**
Model 1	1327	0.480 (0.270–0.689)	0.000 *	672	0.451 (0.160–0.741)	0.002 *	655	0.497 (0.190–0.803)	0.002 *
Model 2	1327	0.446 (0.235–0.657)	0.000 *	672	0.425 (0.136–0.713)	0.004 *	655	0.473 (0.157–0.788)	0.003 *
Model 3	1137	0.308 (0.097–0.519)	0.004 *	576	0.280 (0.002–0.558)	0.050 *	561	0.311 (-0.024–0.647)	0.070
Model 4	1096	0.311 (0.090–0.532)	0.006 *	553	0.268 (−0.030–0.565)	0.080	543	0.290 (−0.032–0.613)	0.080
Model 5	833	0.243 (0.011–0.476)	0.040 *	424	0.236 (−0.056–0.527)	0.110	409	0.257 (−0.106–0.620)	0.160
**Waist to Height Ratio**									
	***n***	**All (*n* = 1327)**	***p* Value**	***n***	**Male (*n* = 672)**	***p* Value**	***n***	**Female (*n* = 655)**	***p* Value**
Model 1	1325	0.006 (0.003–0.009)	0.000 *	672	0.006 (0.001–0.010)	0.012 *	653	0.007 (0.003–0.012)	0.001 *
Model 2	1325	0.006 (0.003–0.009)	0.000 *	672	0.006 (0.002–0.010)	0.008 *	653	0.007 (0.003–0.012)	0.002 *
Model 3	1135	0.005 (0.002–0.008)	0.002 *	576	0.004 (0.000–0.008)	0.070	559	0.006 (0.001–0.011)	0.024 *
Model 4	1094	0.005 (0.002–0.009)	0.001 *	553	0.004 (0.000–0.008)	0.060	541	0.006 (0.001–0.011)	0.031 *
Model 5	831	0.005 (0.001–0.008)	0.009 *	424	0.003 (−0.001–0.008)	0.164	407	0.005 (0.001–0.007)	0.016 *

Model 1: blood Hg levels, Model 2: Model 1 + age + sex, Model 3: Model 2 + seafood consumption, Model 4: Model 3 + blood hematocrit levels+ household income + drinking + smoking, Model 5: Model 4 + exercise. * *p* < 0.05, using general linear regression analysis.

**Table 4:** Crude and Adjusted Odds Ratios (95% Confidence Intervals) of Blood Mercury Level Quartiles to Predict Overweight and the High Waist to Height Ratio (≥0.5) in Korean Adolescents.

**Body Mass Index**										
		**All (*n* = 1327)**	***p***	***p* for Trend**	**Male (*n* = 672)**	***p***	***p* for Trend**	**Female (*n* = 655)**	***p***	***p* for Trend**
Crude		1.32 (1.13–1.55)		0.001 *	1.24 (1.01–1.54)		0.04 *	1.42 (1.12–1.80)		0.004 *
	Q1	Reference			Reference			Reference		
	Q2	0.98 (0.61–1.57)	0.93		1.31 (0.69–2.49)	0.413		0.65 (0.32–1.29)	0.22	
	Q3	1.03 (0.64–1.65)	0.90		0.89 (0.46–1.72)	0.726		1.21 (0.62–2.37)	0.57	
	Q4	2.27 (1.45–3.75)	0.00 *		2.16 (1.17–4.01)	0.015 *		2.40 (1.23–4.69)	0.01 *	
Model 1		1.32 (1.13–1.55)		0.001 *	1.26 (1.02–1.56)		0.03 *	1.42 (1.12–1.80)		0.004 *
	Q1	Reference			Reference			Reference		
	Q2	0.98 (0.61–1.57)	0.93		1.39 (0.72–2.66)	0.327		0.65 (0.33–1.30)	0.23	
	Q3	1.03 (0.64–1.66)	0.89		0.93 (0.48–1.82)	0.833		1.22 (0.63–2.38)	0.56	
	Q4	2.28 (1.45–3.59)	0.00 *		2.28 (1.21–4.27)	0.010 *		2.41 (1.23–4.71)	0.01 *	
Model 2		1.23 (1.03–1.46)		0.02 *	1.21 (0.96–1.52)		0.10	1.27 (0.98–1.65)		0.06
	Q1	Reference			Reference			Reference		
	Q2	1.01 (0.61–1.64)	0.99		1.54 (0.76–3.10)	0.230		0.62 (0.30–1.28)	0.19	
	Q3	0.92 (0.56–1.54)	0.76		0.90 (0.44–1.85)	0.781		1.04 (0.51–2.13)	0.92	
	Q4	1.88 (1.14–3.15)	0.01 *		2.16 (1.06–4.40)	0.033 *		1.70 (0.86–3.72)	0.12	
Model 3		1.22 (1.03–1.46)		0.02 *	1.19 (0.95–1.51)		0.12	1.26 (0.97–1.63)		0.07
	Q1	Reference			Reference			Reference		
	Q2	0.88 (0.48–1.30)	0.99		1.55 (0.77–3.15)	0.221		0.62 (0.84–3.63)	0.13	
	Q3	0.79 (0.48–1.30)	0.75		0.89 (0.44–1.82)	0.749		1.04 (0.51–2.12)	0.92	
	Q4	1.80 (1.10–2.96)	0.01 *		2.11 (1.03–4.31)	0.040 *		1.75 (0.84–3.63)	0.13	
Model 4		1.22 (0.99–1.49)		0.06	1.16 (0.89–1.51)		0.26	1.24 (0.94–1.63)		0.12
	Q1	Reference			Reference			Reference		
	Q2	1.05 (0.59–1.87)	0.87		2.32 (1.04–5.21)	0.04 *		0.46 (0.19–1.13)	0.09	
	Q3	0.79 (0.44–1.42)	0.42		0.70 (0.30–1.62)	0.41		1.01 (0.44–2.32)	0.98	
	Q4	1.93 (1.07–3.46)	0.03 *		2.36 (1.06–5.29)	0.04 *		1.63 (0.76–3.51)	0.21	
**Waist to Height Ratio**										
		**All (*n* = 1296)**	***p***	***p* for Trend**	Male (*n* = 660)	*p*	***p* for Trend**	**Female (*n* = 636)**	***p***	***p* for Trend**
Crude		1.36 (1.09–1.67)		0.005 *	1.34 (1.04–1.72)		0.023 *	1.37 (0.92–2.03)		0.12
	Q1	Reference			Reference			Reference		
	Q2	1.99 (0.96–4.09)	0.063		2.88 (1.17–7.09)	0.022 *		0.96 (0.29–3.13)	0.94	
	Q3	1.67 (0.83–3.39)	0.153		1.99 (0.82–4.85)	0.128		1.21 (0.37–3.92)	0.75	
	Q4	2.98 (1.51–5.90)	0.002 *		3.44 (1.45–8.15)	0.005 *		2.36 (0.77–8.23)	0.13	
Model 1		1.35 (1.09–1.67)		0.005 *	1.36 (1.06–1.75)		0.017 *	1.37 (0.93–2.02)		0.12
	Q1	Reference			Reference			Reference		
	Q2	2.01 (0.97–4.19)	0.061		3.21 (1.28–8.05)	0.327		1.01 (0.31–3.26)	0.99	
	Q3	1.66 (0.81–3.39)	0.166		2.16 (0.88–5,34)	0.833		1.25 (0.38–4.08)	0.71	
	Q4	2.99 (1.49–5.98)	0.002 *		3.73 (1.55–8.97)	0.010 *		2.39 (0.78–7.39)	0.13	
Model 2		1.33 (1.05–1.67)		0.016 *	1.34 (1.04–1.72)		0.024 *	1.36 (0.87–2.12)		0.182
	Q1	Reference			Reference			Reference		
	Q2	2.29 (1.03–5.06)	0.041 *		4.24 (1.56–11.52)	0.005 *		0.86 (0.25–3.04)	0.819	
	Q3	1.56 (0.70–3.48)	0.276		2.34 (0.86–6.4)	0.097		0.96 (0.26–3.52)	0.952	
	Q4	3.06 (1.38–6.80)	0.006 *		4.15 (1.54–11.19)	0.005 *		2.26 (0.68–7.53)	0.182	
Model 3		1.31 (1.04–1.66)		0.021 *	1.31 (1.03–1.68)		0.031 *	1.30 (0.83–2.04)		0.251
	Q1	Reference			Reference			Reference		
	Q2	2.27 (1.03–5.05)	0.043 *		4.36 (1.47–10.77)	0.005 *		0.84 (0.24–2.99)	0.791	
	Q3	1.53 (0.69–3.40)	0.299		2.29 (0.94–6.27)	0.106		0.95 (0.26–3.28)	0.937	
	Q4	2.98 (1.33–6.65)	0.008 *		3.98 (1.47–10.77)	0.007 *		2.01 (0.59–6.92)	0.266	
Model 4		1.26 (0.97–1.66)		0.088	1.22 (0.91–1.63)		0.181	1.36 (0.82–2.25)		0.23
	Q1	Reference			Reference			Reference		
	Q2	2.79 (1.13–6.89)	0.027 *		7.61 (2.13–27.2)	0.002 *		0.59 (0.14–2.60)	0.49	
	Q3	1.23 (0.48–3.41)	0.615		1.74 (0.46–6.57)	0.414		1.15 (0.31–4.34)	0.83	
	Q4	3.00 (1.15–7.84)	0.025 *		4.77 (1.35–16.89)	0.016 *		2.08 (0.53–8.26)	0.29	

Crude: blood mercury level quartiles, Model 1: blood mercury level quartiles + age + sex, Model 2: Model 1 + seafood consumption, Model 3: Model 2 + blood hematocrit levels, Model 4: Model 3 + household income + drinking + smoking + exercise. * *p* < 0.05, using logistic regression analysis.

## 4. Discussion

In the present study, we demonstrated that elevated blood mercury levels were associated with risks for overweight and a high WHtR in Korean children and adolescents after adjusting for possible potential confounders according to data from the KNHANES.

Several studies in adults have shown that the BMI is associated with blood mercury levels; however, they showed inconsistent results [5,8,11]. Additionally, few studies have been performed on children and adolescents, and these studies also showed inconsistent results [11]. One study showed that obesity is associated with blood mercury levels in children and adolescents but had a limitation regarding a lack of adjustment for seafood consumption [26]. In a prospective study, elevated in utero mercury exposure was associated with a higher risk of overweight or obesity in children [27]. Another study in children revealed that the BMI was inversely related to blood mercury levels, suggesting differences in mercury metabolism related to the BMI between adults and children [9]. Our study revealed that blood mercury levels showed a significantly positive association with the BMI after adjusting for covariates, including seafood consumption. Additionally, participants in the highest quartile of blood mercury levels were at a higher risk of overweight than those in the lowest quartile, and the blood mercury levels exhibited acceptable performance in predicting overweight in the ROC analysis. Risk factors known to be associated with overweight and obesity include genetic factors, lifestyle factors, such as caloric intake and physical activity, cultural factors, and environmental factors. The present study suggests that mercury exposure is an additional important risk factor for overweight in Korean children and adolescents. Additionally, blood mercury levels are related to age, the pattern of seafood consumption, genetic factors involved in mercury clearance, and ethnicity [28]. Our study provides additional valuable information on blood mercury levels associated with Korean overweight children and adolescents.

A significant relationship between heavy metal exposure and metabolic syndrome has been demonstrated [29]. Many studies have investigated the role of mercury in metabolic syndrome development, but the findings are inconsistent [30]. In the present study, because of insufficient insulin data, evaluating the relationship between blood mercury levels and metabolic syndrome development was challenging. However, the present study showed that blood mercury levels are significantly associated with the WHtR, which is considered a significant predictor of metabolic syndrome [31]. These findings suggest that mercury exposure has a possible relationship with the development of metabolic syndrome in obese children and adolescents. Additional investigations of the possible effect of mercury exposure on metabolic syndrome should be undertaken.

The most common methods of assessing mercury exposure are urine, blood, and hair testing [32]. The organic mercury content is measured in blood for a recent exposure and hair for a long-term exposure, while urinalysis is used to measure inorganic mercury for the past few months [33]. For most people, an elevated blood mercury level is associated with eating fish and other seafood containing organic mercury. In a study of a general Korean population, seafood intake during the previous 3 days before blood sampling affected blood mercury levels in both male and female individuals [34]. Consistent with these results, our study showed that blood mercury levels were significantly higher in the highest seafood consumption quartile than in the lowest seafood consumption quartile in the previous 24 h. The consumption of fish contaminated with a form of methylmercury is an important exposure pathway in children and adolescents. However, this study is limited in that our data did not include urinary or hair mercury levels to assess long-term mercury exposure. The results of the logistic regression analysis also have limitations, considering the uncertainty of past mercury exposure.

In our study, blood mercury levels were significantly elevated in the overweight male group compared with those in the normal group after controlling for covariates but not in the overweight female group. Additionally, in the logistic regression analysis to predict a high WHtR, the male subgroup showed significant findings but not the female subgroup. Possible explanations are that male individuals have more visceral adipose tissue and less subcutaneous adipose tissue than female individuals and that visceral adipose tissue contains higher toxin levels than subcutaneous adipose tissue [35,36,37]. Our study also showed higher WC in the overweight male group than in the overweight female group. However, no sex differences were demonstrated in the geometric mean value of the blood mercury levels. This finding is consistent with the representative data of blood mercury levels in the Korean general population, including children and adolescents, which revealed sex differences in blood mercury levels in a population older than 20 years [34]. In rodent studies, sex differences in tissue distribution and mercury clearance have been demonstrated, and the findings suggest that these differences may be caused by androgens and estrogen [38,39]. Our result can be explained by sex hormones being absent in participants aged 10–18 years.

Some studies in adults have reported that other factors associated with higher blood mercury levels include household income, alcohol consumption, and smoking [23,40]. In our study, blood mercury levels were not significantly associated with household income, current alcohol consumption or current smoking status after adjustment for covariates. This inconsistency with previous reports could be related to the different definitions of smoking status and alcohol consumption, different populations and small numbers of current alcohol consumers and smokers in our study. In these covariates, smoking is a crucial factor for mercury exposure because the mercury content in tobacco ranges from 2.95 to 10.2 ng of mercury per cigarette [41]. However, in the present study, the number of current smokers in children and adolescents was small; thus, a limitation exists in reflecting the actual effect of mercury exposure on smoking. The hematocrit is also related to the blood mercury level because up to 80% of methylmercury binds to red blood cells [12]. Higher blood hematocrit levels result in more mercury binding in whole blood and a higher mercury concentration in the blood sample [42]. Our results showed that the blood hematocrit levels in male participants were higher than those in female participants. Differences in the hematologic variables between the sexes emerge after the onset of menstruation [43]. Additionally, exercise is a lifestyle factor associated with the risk of overweight or obesity. Our study showed no significant difference in exercise between the normal and overweight groups among all the participants and in the female subgroup but not in the male subgroup. These findings are related to those in a British study, in which male individuals reported more frequent engagement in exercise than female individuals during adolescence [44]. After adjusting for these possible covariates related to blood mercury levels and obesity, we found that blood mercury levels were a significant risk factor for overweight in Korean children and adolescents.

According to the KNHANES 2011–2013, the geometric mean blood mercury level in Korean adults was 3.37 μg/L [5]. These mercury levels are approximately three to four times higher than those of adults in the U.S. (0.86 μg/L), Germany (0.58 μg/L), or Canada (0.76 μg/L) [45,46,47]. A previous study in a general population noted that as the participants became older, the blood mercury levels increased [34]. Our study, conducted in children and adolescents aged 10–18 years, showed that the geometric means of the blood mercury levels were 2.19 μg/L, 2.18 μg/L, and 2.14 μg/L in all, male, and female participants, respectively, after adjusting for covariates. The mercury levels in our study were also much higher than the 0.8 μg/L reference value in German children aged 3–14 years and the 0.3 μg/L median blood mercury level in American children and adolescents aged 1–17 years [48,49]. Accordingly, the blood mercury level in Koreans is higher than that in individuals in Western countries, including children and adolescents. This difference seems to be related to seafood being a staple food in Korea and can be a primary exposure pathway of methylmercury [50]. In our study, the mean intake of seafood was 54.18 g/day, which is higher than that in Germany (37.5 g/day), Canada (24.7 g/day), and the U.S. (12.8 g/day) [51,52,53].

Many types of fish do not contain high levels of mercury [54]. While balancing concerns for methylmercury exposure, eating different types of fish benefits nutrition because fish contain high-quality protein and omega-3 fatty acids. According to the Environmental Protection Agency (EPA)-FDA recommendation in the U.S., women of childbearing age (aged approximately 16–49 years), pregnant and breastfeeding women, and young children should eat more fish that are low in mercury for important developmental and health benefits [55]. They recommend that those people eat two to three servings (8–12 ounces for adults and children older than 10 years) of various fish and shellfish each week. Mercury has been widely recognized as a threat to the health of children and adolescents, and a global policy regarding seafood consumption is needed to reduce and prevent mercury exposure.

This cross-sectional study has a limitation in that establishing accurate cause-effect relationships between overweight, a high WHtR, and blood mercury levels is challenging. Blood mercury levels are influenced by the elapsed time between the individual’s exposure and specimen collection [15]. These nationwide cross-sectional data, including anthropometric measures, other variables, and blood mercury concentrations, were collected simultaneously without considering the elapsed time. Thus, difficulty in interpreting whether blood mercury concentrations reflect actual mercury exposure may result. To understand the underlying pathogenesis of this association between overweight, a high WHtR, and blood mercury levels, further studies are required. Additionally, because environmental conditions are changing, reference values should be continuously checked and updated when new information becomes available. Recent national data for mercury exposure in the general population are needed.

## 5. Conclusions

Our study demonstrated an association of mercury exposure with the risks of overweight and a high WHtR in Korean children and adolescents. These findings suggest the need to screen blood mercury levels in obese children and adolescents in practice and establish global guidelines for seafood consumption to reduce mercury exposure.

## Data Availability

Supporting data can be obtained from the corresponding author upon reasonable request.
